# Effect of the synthetic cannabinoid HU-210 on quorum sensing and on the production of quorum sensing-mediated virulence factors by *Vibrio harveyi*

**DOI:** 10.1186/s12866-015-0499-0

**Published:** 2015-08-12

**Authors:** Divya Soni, Reem Smoum, Aviva Breuer, Raphael Mechoulam, Doron Steinberg

**Affiliations:** Biofilm Research Laboratory, Institute of Dental Sciences, Faculty of Dental Medicine, Hebrew University-Hadassah Medical Center, Jerusalem, Israel; Institute for Drug Research, Faculty of Medicine, Hebrew University, Jerusalem, Israel

## Abstract

**Background:**

Bacterial populations communicate through the cell density-dependent mechanism of quorum sensing (QS). *Vibrio harveyi*, one of the best studied model organisms for QS, was used to explore effects of the synthetic cannabinoid HU-210 on QS and different QS-regulated physiological processes in bacteria.

**Results:**

Analysis of QS-regulated bioluminescence in wild-type and mutant strains of *V. harveyi* revealed that HU-210 affects the autoinducer-2 (AI-2) pathway, one of three known QS cascades of *V. harveyi*. Furthermore, QS-mediated biofilm formation and swimming motility in the mutant strain BB152 (AI-1^−^, AI-2^+^) were significantly reduced in the presence of HU-210. HU-210 inhibited QS-mediated virulence factor production without any inhibitory effect on bacterial growth. It also alters the expression of several genes, which are regulated by QS, specifically downregulating the genes of the AI-2 QS cascade.

**Conclusion:**

First evidence is being provided for interference of bacterial signal-transduction systems by a synthetic cannabinoid. The effect of HU-210 was specific to the AI-2 cascade in *V. harveyi*. AI-2 is known as a "universal autoinducer" and interference with its activity opens a broad spectrum of applications for synthetic cannabinoids in future research as a potential anti-QS agent.

**Electronic supplementary material:**

The online version of this article (doi:10.1186/s12866-015-0499-0) contains supplementary material, which is available to authorized users.

## Background

Bacteria communicate and coordinate population behavior through the mechanism of quorum sensing (QS), which controls the expression of genes that affect a variety of bacterial processes [1, 2]. QS is based on small signaling molecules, termed autoinducers (AIs), which control factors such as bioluminescence, pigment production, motility and biofilm formation, among many others [3–5]. The QS, free-living marine bacterium *Vibrio harveyi* produces and responds to at least three distinct AIs [6]: HAI-1, an acyl homoserine lactone [7]; AI-2, a furanosyl-borate-diester [8]; and CAI-1, a long-chain amino ketone (Z)-3-aminoundec-2-en-4-one (Ea-C8-CAI-1) [9]. AI-2 is referred to "universal autoinducer" as it is found in numerous Gram-positive and Gram-negative bacteria [10, 11]. A better understanding of QS actions and novel ways to affect them is providing an opportunity to manipulate bacterial properties that are of major importance in industry, agriculture and medicine.

Plant and endogenous cannabinoids have been investigated extensively, particularly as agonists of the endocannabinoid signaling system and as therapeutically active substances [12, 13]. Both delta-9-tetrahydrocannabinol (THC), the major psychoactive constituent of cannabis [14] and cannabidiol (CBD), a non-psychoactive constituent, alter immune functions, mostly via suppression. The mechanism governing these activities has not been fully elucidated (for a recent review see Cabral et al. [15]). In most investigations, these phytocannabinoids have been found to enhance the susceptibility of mice to microorganisms (both bacteria and viruses). However, several reports have indicated that administration of THC leads to lower viral loads and moderation of disease progression [15].

**Fig. 1:**
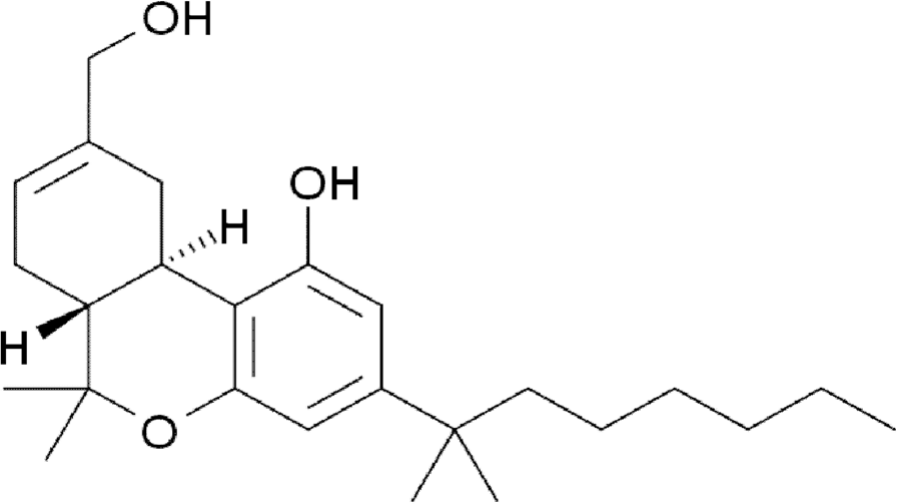
Chemical structure of the synthetic cannabinoid HU-210, used in this study

The effect of cannabinoids on bacterial QS has never been investigated. We chose to study the activity of the synthetic cannabinoid HU-210 (Fig. 1), because its physical and chemical properties have been well defined. HU-210 was first synthesized from (1R,5S)-myrtenol [16]. It displays a multiplicity of biochemical, pharmacological, and behavioral effects, most of which depend on its potent agonistic activity at the cannabinoid receptors CB1 and CB2 [17].

## Results

### Effect of HU-210 on *V. harveyi* bioluminescence

**Fig. 2:**
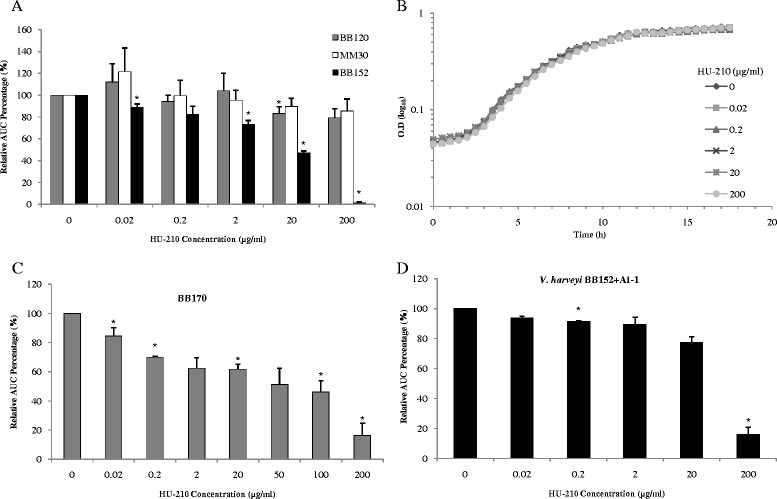
Effect of HU-210 on growth and bioluminescence production of *V. harveyi*. (**a**) Comparison of relative bioluminescence production by *V. harveyi* BB120 (wild type), MM30 (AI-1^+^, AI-2^−^) and BB152 (AI-1^−^, AI-2^+^) with different HU-210 concentrations, presented as area under the curve. (**b**) Growth curve of *V. harveyi* BB152 with different HU-210 concentrations. (**c**) Relative bioluminescence production by *V. harveyi* BB170 (Sensor-1^−^, Sensor-2^+^) with different HU-210 concentrations. (**d**) Relative bioluminescence production by *V. harveyi* BB152 with different HU-210 concentrations and simultaneous supplementation with exogenous AI-1 isolated from *V. harveyi* MM30 (AI-1^+^, AI-2^−^), presented as area under the curve. Presented data are means and SD of three independent experiments, each performed in triplicate.*P < 0.05 compared with control

To investigate the effect of HU-210 on the QS cascade of *V. harveyi* wild-type strain BB120 and QS-mutant strains (Table 1), bioluminescence was measured with and without HU-210 in the growth media. First, we determined whether addition of HU-210 to the growth media alters the bioluminescence of the wild-type *V. harveyi* strain. Results showed no significant difference in bioluminescence between samples with and without HU-210 (Fig. 2a). The experiments were repeated with other *V. harveyi* strains mutated in the QS cascade. Addition of HU-210 to the growth media resulted in an up to 98 % decrease in the bioluminescence of *V. harveyi* mutant BB152 (AI-1^−^, AI-2^+^), in a dose-dependent manner, whereas bioluminescence of the mutant strain MM30 (AI-1^+^, AI-2^−^) was not significantly affected (Fig. 2a). However growth of *V. harveyi* mutant BB152 (Fig. 2b) and other mutant strains (data not shown) was not affected by HU-210. Results indicated that a notable effect of HU-210 was specific to AI-2 in the QS cascade of *V. harveyi*, as this AI regulates QS of *V. harveyi* mutant strain BB152.

**Table 1:** *V. harveyi* strains used in this study

*V. harveyi* strain	Relative genotype	Relative phenotype
BB120	Wild type	AI-1^+^, AI-2^+^; sensor-1^+^, sensor-2^+^
BB152	(*BB7* or *BB120*) *luxLM*::Tn5	AI-1^−^, AI-2^+^; sensor-1^+^, sensor-2^+^
MM30	(*BB120*) *luxS*::Tn5	AI-1^+^, AI-2^−^; sensor-1^+^, sensor-2^+^
MM77	*luxLM*::Tn5, *luxS*::Tn5	AI-1^−^, AI-2^−^; sensor-1^+^, sensor-2^+^
BB170	*luxN*::Tn5	AI-1^+^, AI-2^+^; sensor-1^−^, sensor-2^+^

To further analyze the mode of action of HU-210, another mutant strain, BB170 (Sensor-1^−^, Sensor-2^+^) was tested. Bioluminescence in *V. harveyi* BB170 is mainly controlled by AI-2, as this strain is not responsive to AI-1 stimulation due to the absence of sensor-1 [18]. In the presence of HU-210, bioluminescence production by mutant strain BB170 was decreased by up to 85 %, further confirming the specific action of HU-210 on AI-2 (Fig. 2c). To validate the molecular QS target of HU-210, another experiment was designed in which mutant strain MM77 (AI-1^−^, AI-2^−^), in the absence or presence of different doses of HU-210, was simultaneously incubated with 10 % (v/v) spent medium containing exogenous AI-1 and AI-2 isolated from *V. harveyi* MM30 (AI-1^+^, AI-2^−^) and BB152 (AI-1^−^,AI-2^+^) respectively. The inhibitory effect of HU-210 on bioluminescence production was more pronounced in samples to which exogenous AI-2 had been added (~95 % decrease), suggesting that the effect of HU-210 is not only on the production of AI-2 signal, but also on the reception of those signal molecules by hybrid sensor kinases present in mutant strain MM77 (Additional file 1: Figure S1).

Interestingly when mutant strain BB152 (AI-1^−^, AI-2^+^) was supplemented with exogenous AI-1 from MM30 (AI-1^+^, AI-2^−^) and simultaneously treated with different concentrations of HU-210, the latter's dose-dependent inhibitory effect could be reversed and bioluminescence level of this mutant strain restored to the similar level of the wild-type BB120, except in sample with highest HU-210 concentration (200 μg/ml) (Fig. 2d).

### Effect of HU-210 on *V. harveyi* biofilm biomass and DNA quantity

The effect of HU-210 on other QS-controlled processes in *V. harveyi*, such as biofilm formation, was also tested. Biofilms were grown with and without different concentrations of HU-210 using different *V. harveyi* strains (Table 1). The biofilm biomass formed by the different strains was quantified using crystal violet (CV) staining. The effect of HU-210 on biofilm biomass formed by wild-type BB120 and mutant strain MM30 was minimal, as no significant decrease was observed with increasing concentrations of HU-210 (Additional file 1: Figure S2). Consistent with these results, an up to 80 % decrease was observed in biofilm biomass of mutant strain BB152 (AI-1^−^, AI-2^+^). Inhibition of biofilm biomass formation by HU-210 in mutant BB152 was found to be dose-dependent (Additional file 1: Figure S2).

**Fig. 3:**
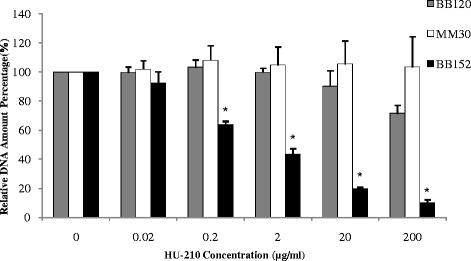
DNA quantification by qPCR. Comparison of quantified DNA of *V. harveyi* BB120 (wild type), MM30 (AI-1^+^, AI-2^−^) and BB152 (AI-1^−^, AI-2^+^) biofilm formed with or without different concentrations of HU-210. Presented data are means and SD of three independent experiments, each performed in triplicate.*P < 0.05 compared with control

This anti-biofilm effect was further confirmed using a DNA-quantification method which specifically quantified the DNA present in different *V. harveyi* biofilms. DNA quantification results showed the same trend of inhibitory action for HU-210: biofilm in the mutant strain BB152 was dose-dependently inhibited by up to 80 % (Fig. 3), whereas there was no significant decrease in the amount of DNA present in wild-type BB120 and mutant MM30 biofilms.

### Biofilm depth, bacterial viability and presence of extracellular polysaccharides (EPS)

**Fig. 4:**
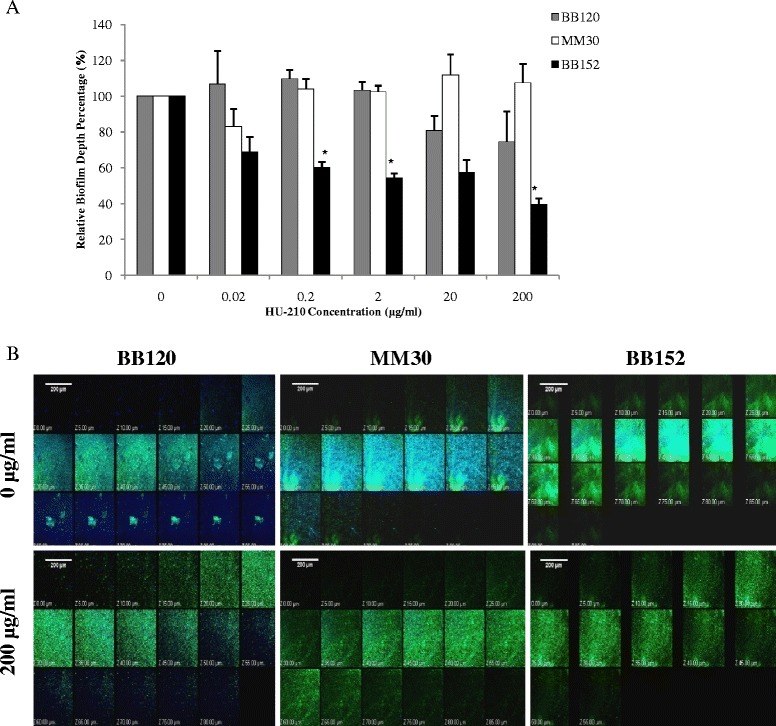
Analysis of *V. harveyi* biofilms by CLSM. (**a**) Comparison of biofilm depth of *V. harveyi* BB120 (wild type), MM30 (AI-1^+^, AI-2^−^) and BB152 (AI-1^−^, AI-2^+^) formed with or without different concentrations of HU-210. Presented data are means and SD of three independent experiments.*P < 0.05 compared with control. (**b**) *V. harveyi* BB120 (wild type), MM30 (AI-1^+^, AI-2^−^) and BB152 (AI-1^−^, AI-2^+^) biofilms grown with or without HU-210 (200 μg/ml) and stained with SYTO 9 dye (green), which stains live bacteria, and with concanavalin A–Alexa Fluor 647 conjugates (blue), which stain extracellular polysaccharides. Biofilm depth of *V. harveyi* BB120 (wild type), MM30 (AI-1^+^, AI-2^−^) and BB152 (AI-1^−^, AI-2^+^) biofilms in control (without HU-210) is 85 μm, 80 μm ,95 μm and in samples with HU-210 (200 μg/ml) is 80 μm, 85 μm, 55 μm respectively

To further evaluate the effect of HU-210 on biofilm formation by *V. harveyi*, we analyzed the morphology of the biofilms generated by the *V. harveyi* wild type BB120, and mutants MM30 (AI-1^+^, AI-2^−^) and BB152 (AI-1^−^, AI-2^+^) using confocal laser scanning microscopy (CLSM). The depth of the biofilms formed by the wild type and mutant strain MM30 was least affected by the addition of HU-210. However biofilms formed by mutant BB152 in control samples (without HU-210) were more confluent (95 μm) than those formed in the presence of HU-210 (55 μm), and showed a gradual decrease in average depth with increasing concentrations of HU-210 (Fig. 4a & b).

The amount of EPS was normalized to live cells in the biofilm, and we observed less EPS in biofilms formed by all tested *V. harveyi* strains in the presence of HU-210 vs. controls. However, the decrease was only significant for mutant strain BB152, where up to 75 % less EPS was measured in the presence of 200 μg/ml HU-210 vs. controls. Bacterial cell viability in the biofilms was not significantly affected by HU-210 in any of the tested *V. harveyi* strains (Table 2).

**Table 2:** Analysis of biofilms formed by *V. harveyi* strains in the presence/absence of HU-210

*V. harveyi* strains	Flourescence Intensity	
with/without HU-210	Live/Dead	Relative EPS/Live Percentage
BB120	2.40 ± 1.23	100 ± 0
BB120 + 200 μg/ml HU-210	2.80 ± 1.13	45.98 ± 8.71
MM30	1.60 ± 0.99	100 ± 0
MM30 + 200 μg/ml HU-210	2.81 ± 1.15	88.75 ± 2.94
BB152	1.89 ± 0.69	100 ± 0
BB152 + 200 μg/ml HU-210	3.63 ± 0.03	25.50 ± 4.50*

Based on data obtained from CLSM. Fluorescence intensity was measured using Image J software. Presented

data are means and SD of three independent experiments.*P < 0.05

### Effect of HU-210 on swimming motility of *V. harveyi*

**Fig. 5:**
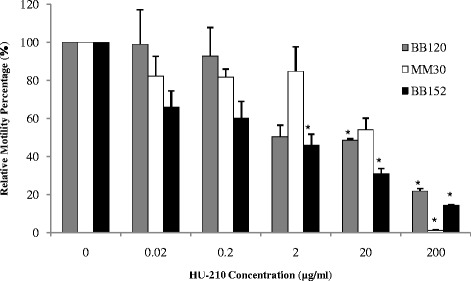
Comparison of swimming motility of *V. harveyi* BB120 (wild type), MM30 (AI-1^+^, AI-2^−^) and BB152 (AI-1^−^, AI-2^+^) with different concentrations of HU-210 quantified using Image J Software. Presented data are means and SD of two independent experiments (*P < 0.05)

Swimming motility assays were performed with wild-type strain BB120 and mutant strains MM30 and BB152. A significant decrease (*P* < 0.05) in swimming motility was observed in all three strains in the presence of the highest tested concentration of HU-210 (200 μg/ml). This effect of HU-210 was most evident in mutant strain BB152 (AI-1^−^, AI-2^+^) as it interfered with swimming motility at lower concentrations as well (above 2 μg/ml) (Fig. 5). In both the wild-type strain BB120 and the mutant strain BB152, the observed reduction in swimming motility was dose-dependent (up to 80 % and 85 %, respectively), whereas in mutant strain MM30, up to 95 % inhibition was observed at the highest HU-210 concentration tested (200 μg/ml).

### Effect of HU-210 on mRNA levels of different genes involved in QS regulation

The molecular basis of the inhibitory action of HU-210 on QS and biofilm formation in *V. harveyi* mutant strain BB152 was investigated. We quantified mRNA levels of six genes which had been previously found to be regulated by the AI-1- and AI-2-dependent QS in *Vibrio*. Genes specific to the AI-2 QS cascade—*luxS*, *luxP* and *luxQ*—were downregulated in the presence of 2 μg/ml HU-210 in wild-type BB120 and mutant BB152, whereas genes specific to the AI-1 cascade—*luxM* and *luxN*—were upregulated in wild-type BB120 and mutant strain MM30. Significant downregulation of AI-2 synthase *luxS* and master regulator *luxR* was only observed in mutant strain BB152 treated with HU-210 (Table 3). Results suggested that HU-210 can inhibit QS through the AI-2 cascade by inhibiting the production of AI-2 signal molecules through *luxS*. Furthermore, decreased expression of *luxP* and *luxQ* in mutant strain BB152 also suggested that the mechanism of action proceeds through these hybrid sensor kinases. As an outcome, there was less expression of *luxR*—the master regulator of QS target genes—resulting in suppressed activity of the AI-2-mediated QS cascade present in mutant strain BB152. This could also explain the specific inhibitory effect of HU-210 on bioluminescence, biofilm-forming ability and swimming motility of mutant strain BB152 observed throughout the study.

**Table 3:** Relative mRNA expression of different genes in *V. harveyi*

Relative mRNA expression	BB120	MM30	BB152
with HU-210 (2 μg/ml)	RQ value		
*luxS*	0.81 ± 0.03	-	0.42 ± 0.05*
*luxP*	0.98 ± 0.45	-	0.37 ± 0.25
*luxQ*	1.1 ± 0.65	-	0.36 ± 0.11
*luxM*	3.05 ± 0.46	4.04 ± 1.42	-
*luxN*	1.31 ± 0.31	2.34 ± 0.87	-
*luxR*	0.45 ± 0.12	1.34 ± 0.19	0.43 ± 0.11*

Relative expression of genes was measured using real-time RT-PCR as described in Materials and Methods. Fold change of the transcriptional level was calculated using ABI-Prism 7300 software v1.1 with RQ study 1.0, Applied Biosystems. The expression results represent mean ± SD of two independent experiments. *P < 0.05

## Discussion

Bacteria communicate via QS using extracellular chemical signaling molecules. AI-2 used for QS signaling is found in both Gram-positive and Gram-negative bacteria. Recent studies have focused on finding small molecules analogous to AIs which can alter QS-regulated pathogenic behaviors and biofilm formation in bacteria [19]. The action of cannabinoids on microbes and viruses has been documented [15]. However their effect on bacterial QS pathways is unknown.

*V. harveyi* is one of the best-characterized model organisms for the study of QS [20, 21]. Therefore, we investigated the effect of the potent synthetic cannabinoid HU-210 on QS-regulated bioluminescence, biofilm formation and swimming motility of *V. harveyi* strains*.* Further research on cannabinoid–bacteria interactions will involve exploring more derivatives and their effects on other types of bacteria.

Our study focused mainly on the effect of HU-210 on QS-mediated pathways at concentrations below the minimum inhibitory concentration (MIC). Growth of *V. harveyi* strains was unaffected at all tested concentrations (0.2–200 μg/ml) of HU-210. In experiments with *V. harveyi* wild-type strain BB120 and mutant strains BB152 and MM30, HU-210 only significantly reduced bioluminescence of the mutant strain BB152. Strain BB152 is a HAI-1 synthase mutant and inhibition of its bioluminescence by HU-210 led us to postulate that this synthetic cannabinoid acts on the AI-2 mediated QS cascade. Using the bioluminescence reporter strain BB170 (Sensor-1^−^, Sensor-2^+^), we were able to address the specific inhibitory action of HU-210 on AI-2. The mutant BB152 (AI-1^−^, AI-2^+^) was incubated simultaneously with different doses of HU-210 and exogenous AI-1 and assessed for further effects of HU-210. Interestingly, exogenous AI-1 reversed HU-210 inhibition, indicating complementation by exogenous AI-1 for the dose-dependent effect of HU-210, except in samples with the highest tested HU-210 concentration (200 μg/ml). This could be because at the highest concentration (200 μg/ml), HU-210 strongly inhibited AI-2 activity (~95 % decrease) in BB152 and the observed bioluminescence production was due only to the excess exogenous AI-1 available at the time.

The plant kingdom is a well-known source of medicines, including numerous antimicrobial agents. Several other quorum-quenching molecules have been isolated from plants [22]. For example, the plant constituent curcumin was recently shown to interfere with QS-mediated bioluminescence production in *V. harveyi* [23]. Similarly, extracts of garlic have been shown to block QS of *Pseudomonas aeruginosa* [24], and vanilla extracts are capable of interfering with QS in *Chromobacterium violaceum* [25].

Previous studies have confirmed that biofilm formation is one of the primary attributes toward bacterial pathogenicity and that it is partially controlled by QS [26]. Results of the present study suggest that HU-210 at the tested concentrations significantly inhibited the biofilm-forming capability of *V. harveyi* mutant strain BB152 (AI-1^−^, AI-2^+^). The amount of total biomass in the biofilm, determined by CV staining and DNA quantification, decreased with increasing concentration of HU-210. Since the inhibitory action of HU-210 was more prominent in the mutant strain BB152 (AI-1^−^, AI-2^+^) than in the other tested *V. harve*yi strains, HU-210 seems to interfere with the AI-2-dependent QS pathway, resulting in less QS and ultimately less biofilm formation. Results of the current study are consistent with previous observations made in *Vibrio* spp. wherein a pytochemical, resveratrol significantly reduced total biofilm bimoass [27].

The anti-biofilm effect of HU-210 was further confirmed by CLSM observation of biofilm architecture, showing a significant decrease in the depth of the biofilm formed by mutant BB152; however, there was no such effect on biofilm formed by wild-type strain BB120 or mutant strain MM30. The average thickness of the biofilm formed by these strains remained almost the same in HU-210-treated and untreated samples. The substantial reduction in biofilm thickness for strain BB152 was comparable to the result of a study in which S6-15, an extract from the marine bacterium *Bacillus pumilus*, was shown to be effective against biofilm formation in a wide range of Gram-positive and Gram-negative bacteria [28].

Bacterial motility is an important virulence factor in many pathogens. Some *Vibrio* spp. (including *V. harveyi*) possess dual flagellar systems that are suited to movement under different conditions [29]. A recent study reported that QS enhances flagellar motility in pathogenic *V. harveyi* [30]. To further investigate the effect of HU-210 on QS, we checked its possible impact on swimming motility of *V. harveyi*. The results indicated an inhibitory effect of HU-210 on QS via its restrictive effect on the motility of the wild type as well as mutant strains BB152 and MM30. Observations from this study fall in line with another study in which curcumin was shown to affect swimming motility of *V. harveyi* [23].

Biofilm formation is a developmental process due to changes in the structural and regulatory genes required at various steps of its formation [31–33]. To unveil the molecular basis of *V. harveyi* BB152 bioluminescence and biofilm inhibition by HU-210, we measured differential expression of genes regulating QS-phenotypes in the presence and absence of HU-210. In *V. harveyi*, the AI-2-mediated QS system is composed of LuxS, LuxP and LuxQ. LuxS is responsible for the synthesis of AI-2 [8]. LuxP is a periplasmic protein that binds AI-2, and LuxQ is a hybrid sensor kinase. Lux R is the master regulator of all three parallel QS systems in *V. harveyi* [34]. We observed less expression of *luxS, luxP, luxQ* and *luxR* in mutant strain BB152 treated with HU-210, suggesting that this cannabinoid interferes with AI-2 production, as well as with reception of these QS signals by sensor kinases, resulting in lower activity of LuxR and less activation of QS target genes. This notion is in agreement with several other reports showing disruption of QS-regulated gene expression in *V. harveyi* by halogenated furanone [35, 36].

It has been reported that three QS systems work in parallel, based on a coincidence detection scheme, to regulate different genes' expression in *V. harveyi* [4]. We noticed that *luxM* (AI-1 producer) and *luxN* (hybrid sensor kinase which perceives AI-1) in wild-type BB120 as well as in mutant strain MM30 were upregulated in the presence of 2 μg/ml HU-210. It has been recently shown that in a growing wild-type *V. harveyi* culture, AI-2 synthesis is quickly followed by that of HAI-1 [21]. Based on this knowledge, it becomes evident that the observed limited effect of HU-210 on wild-type BB120 bioluminescence and biofilm formation is a result of the coincidence detection scheme in which AI-1 of BB120 (AI-1^+^, AI-2^+^) complements the QS system when AI-2 activity is interfered with by HU-210.

The present study is the first report on the effect of a cannabinoid on QS-mediated processes, namely bioluminescence, biofilm formation and swimming motility of a bacterial species. The inhibitory action of HU-210 was found to proceed through AI-2 and it was the result of altered expression of genes involved in the AI-2 QS cascade. AI-2 is known as the "universal autoinducer" and interference in its activity opens a broad spectrum of applications for synthetic cannabinoids in future research with prokaryotes. Further investigations need to be carried out to establish the molecular aspects and mode of action of cannabinoids' effects on QS.

## Conclusions

The synthetic cannabinoid HU-210 has an inhibitory effect on QS and QS-dependent properties, such as bioluminescence, biofilm formation and swimming motility of *V. harveyi*, without affecting its growth. Furthermore, HU-210's effect was found to proceed through the AI-2-mediated QS cascade. Results of the present study suggest a novel mode of action for synthetic cannabinoids on bacteria via the bacterial communication system, and potential use of cannabinoids as anti-QS agents.

## Methods

### Bacterial strains and growth

*V. harveyi* wild-type and mutant strains (Table 1) were grown aerobically overnight at 30 °C in AB-medium. Bacterial strains were kindly provided by B. Bassler (Princeton University).

### HU-210 preparation

HU-210 was synthesized as described previously [37]: m.p. 140–1 °C (from pentane), [α]_D_ -227 (CHCl_3_), δ (CDCl_3_) 6.40, 6.25 (2 arom H's), 5.6 (C = CH), 4.09 (CH_2_–O). Stock solutions were prepared by dissolving 5 mg HU-210 in 250 μl of 100 % ethanol and stored at 4 °C until use. Working concentrations were prepared from the stock solutions by diluting with sterile AB-medium to final concentrations of 0.2–200 μg/ml.

### MIC determination

*V. harveyi* wild-type strain BB120 was grown at 30 °C in the presence of HU-210 at concentrations ranging from 2–4 mg/ml in 96-well transparent plates (Nunc, Roskilde, Denmark). After 24 h of incubation, bacterial growth was determined by absorbance at 595 nm (OD_595_) (TECAN GENios reader, Schweiz, Austria). The MIC was recorded as the minimum concentration that exhibited visible growth inhibition and all further experiments were performed at sub-MIC [38].

### Bioluminescence assay

QS regulates bioluminescence in *V. harveyi* [39]. For the bioluminescence assay, *V. harveyi* overnight culture (O.D_595_ ~ 0.7) was diluted 1:200 in fresh AB-medium along with different concentrations of HU-210. After dilution, 180 μl bacterial culture was transferred to 96-well white plates with an optic bottom (Nunc) and the readings were taken using the GENios reader at 30 °C. Luminescence was measured every 30 min in parallel with absorbance measurement (OD_595_) for 18 h. Relative luminescence unit (the amount of luminescence per unit of growth) was calculated as the quotient of luminescence value and growth for each well. The area under the curve was calculated for each sample, compared with a control sample (without HU-210) and reported as a percentage [38, 40].

### Biofilm biomass quantification using the CV method

Biofilm biomass was quantified by CV staining as described previously [41] with slight modifications. Briefly, a 96-well polystyrene plate (Nunc) was seeded with 20 μl of *V. harveyi* overnight culture (O.D_595_ ~ 0.7) followed by addition of 160 μl AB-medium and 20 μl of HU-210 at different concentrations for biofilm growth. The plate was incubated for 24 h at 30 °C. Generated biofilms were washed carefully twice using 200 μl saline solution. The biofilms were stained with 200 μl of 0.1 % (w/v) CV (Merck, Darmstadt, Germany) solution for 20 min, then washed twice with saline and the stained biofilms were dried overnight at room temperature. Next, 33 % acetic acid was added to elute the CV for 60 min along with shaking at room temperature. The extract was transferred to a new 96-well plate and OD_595_ was measured using the GENios reader.

### DNA quantification by quantitative (q) PCR

For biofilm growth, a polystyrene 48-well plate (Nunc) was seeded with 50 μl of *V. harveyi* overnight culture (O.D_595_ ~ 0.7) followed by addition of 400 μl AB-medium and 50 μl HU-210 at different concentrations. The plate was incubated for 24 h at 30 °C. Generated biofilms were washed carefully twice using 500 μl of saline solution.

DNA extraction and quantification were performed as described previously [42, 43]. Briefly, 160 μl NaOH (0.05 M) (Bio Lab Ltd., Jerusalem, Israel) and 40 μl DEPC-treated water (Bio Basic Canada Inc., Markham, Ontario, Canada) were added to each well. The plate was then immersed in a hot water bath for 1 h at 60 °C. Then, 18.5 μl Tris buffer (pH 7) (Eastman Kodak Company, Rochester, NY, USA) was added to each well and extracted DNA samples were stored at −20 °C until further use.

DNA samples from different *V. harveyi* biofilms were quantified by qPCR with specific primers for 16S rRNA using an ABI prism instrument (Applied Biosystems Prism 7300, Foster City, CA, USA). The amount of DNA was quantified according to the specific standard curve. Total genomic DNA was extracted from an overnight culture of *V. harveyi* BB120 using GenElute Bacterial Genomic DNA kit (Sigma Aldrich, St. Louis, MO, USA) as per the manufacturer’s instructions. Extracted genomic DNA for the standard curve analysis was stored at −20 °C.

### Biofilm depth, cell viability and presence of EPS

Bacterial cell viability, presence of EPS and depth of the biofilms grown as described above were examined by CLSM (Olympus Fluoview 300, Tokyo, Japan) with a UPLSA 10X/0.4 lens. The biofilm was formed by seeding 20 μl *V. harveyi* overnight culture (O.D_595_ ~ 0.7), 160 μl AB- medium and 20 μl of HU-210 at different concentrations in a 96-well plate (Nunc). The plate was incubated for 24 h at 30 °C under aerobic conditions. Biofilms were then washed carefully with saline solution and stained for 20 min at room temperature with different stains. Biofilms were washed again with saline solution before visualization under the microscope.

To examine cell viability, biofilms were stained with LIVE/DEAD BacLight fluorescent dye (Life Technologies, Carlsbad, CA, USA). SYTO9 fluorescence was measured using 488 nm excitation and 515 nm emission filters, and propidium iodide (PI) fluorescence was measured using 543 nm excitation and 570 nm emission filters (Olympus). EPS were stained with concanavalin A–Alexa Fluor 647 conjugates (Invitrogen Molecular Probes, Carlsbad, CA, USA). The biofilm depth was determined by acquiring optical sections at 5-μm spacing using a previously described method [31]. Biofilm depth was examined at the center of each well and quantified as the distance between the highest and lowest section. Fluorescence intensity per unit area was calculated using Image J software (National Institutes of Health) for each color separately.

### Swimming motility assay

The swimming motility assay was performed on soft agar plates using a previously described method [30] with some modifications. Briefly, AB-medium with 0.2 % (w/v) agar was prepared and autoclaved. After cooling to 60 °C, different concentrations of HU-210 were added and the medium was poured into small petri dishes. After solidification, 3 μl *V. harveyi* overnight culture (O.D_595_ ~ 0.5) was inoculated at the center of the agar. Agar plates without HU-210 served as controls. The plates were incubated at 30 °C for 15 h, then the area of the motility halo was measured using Image J software and compared with controls.

### RNA extraction

*V. harveyi* wild-type BB120 and mutant strains MM30 and BB152 were grown overnight planktonically with and without (control) 2 μg/ml HU-210. *V. harveyi* overnight culture (200 μl; O.D_595_ ~ 0.7) was added in triplicate to a 12-well polystyrene plate (Nunc) along with 1.6 ml AB-medium and 200 μl HU-210 (2 μg/ml). The plate was incubated for 24 h at 30 °C under aerobic conditions. Following incubation, the bacterial culture in each well was mixed vigorously and transferred to 10-ml tubes. Further RNA extraction was performed using a previously described method [32]. Briefly, 2 ml of RNA Protect (Qiagen, Hilden, Germany) was added to each tube, mixed well and incubated at room temperature for 5 min. Cells were harvested and DNA-free RNA was isolated using the RNeasy MINI kit (Qiagen) according to the manufacturer’s instructions. RNA purity and quantity were determined using Nanodrop (Nanovue, GE Healthcare Life Sciences, Buckinghamshire, UK). RNA integrity was determined using a bioanalyzer (Agilent, Santa Clara, CA, USA) and the samples were stored at −80 °C for later use.

### Reverse transcription (RT) and real-time PCR

Reverse transcription was performed with the SuperScript™ First-Strand Synthesis Kit (Invitrogen, Life Technologies) in accordance with the manufacturer’s instructions.

The synthesized cDNA was used for real-time PCR analysis with specific primers for the genes examined in this study. The RT-PCR was performed as described previously [44]. The PCR thermal profile included an initial denaturation at 95 °C for 10 min, followed by a 40-cycle amplification consisting of denaturation at 95 °C for 15 s and annealing and extension at 60 °C for 1 min.

As an additional control for each primer pair and each RNA sample, the cDNA synthesis reaction was performed in the absence of reverse transcriptase in order to check for contamination with residual genomic DNA. The expression levels of all genes tested by real-time PCR were normalized to the 16S rRNA gene of *V. harveyi* as an internal standard. There was no significant difference in the expression of the 16S rRNA gene for the two conditions and samples. Fold change of transcription level was calculated using ABI Prism 7300 SDS Software v1.1 with RQ Study 1.0 (Applied Biosystems) [43, 45].

### Statistical analysis

Experiments were performed independently three times in triplicate and the data were analyzed statistically using Student's t test, with a P value of less than 0.05 considered significant.

## Additional file

Additional file 1: Figure S1. Comparison of relative bioluminescence production by *V. harveyi* MM77 (AI-1^-^, AI-2^-^, sensor-1^+^, sensor-2^+^) with different HU-210 concentrations when simultaneously supplemented with exogenous AI-1 and AI-2 from *V. harveyi* mutant strains MM30 (AI-1^+^, AI-2^-^) and BB152 (AI-1^-^, AI-2^+^) respectively, presented as area under the curve. Presented data are means and SD of three independent experiments, each performed in triplicate. *P < 0.05. **Figure S2**. Biofilm biomass quantification of *V. harveyi* wild type BB120, mutant strain MM30 and BB152 biofilms using CV staining. The staining strength is an indication of the amount of biofilm mass formed in AB –media with/without different concentrations of HU-210. Graph represents calculation of biofilm biomass relatively with control. Presented data are means and SD of three independent experiments, each performed in triplicate. *P < 0.05 (DOC 827 kb)

## Abbreviations

QS: Quorum sensing
AI: Autoinducer
CB1: Cannabinoid receptor type-1
CB2: Cannabinoid receptor type-2
OD: Optical density
RT: Reverse transcription
PCR: Polymerase chain reaction
qPCR: Quantitative real-time PCR
CV: Crystal violet
