# Supplementary material for: Effect of the synthetic cannabinoid HU-210 on quorum sensing and on the production of quorum sensing-mediated virulence factors by Vibrio harveyi
Source: BMC Microbiol. 2015 Aug 12;15:159. doi: 10.1186/s12866-015-0499-0 (PMC4531395; doi:10.1186/s12866-015-0499-0)
Supplement: Additional file 1: Figure S1. — Comparison of relative bioluminescence production by V. harveyi MM77 (AI-1-, AI-2-, sensor-1+, sensor-2+) with different HU-210 concentrations when simultaneously supplemented with exogenous AI-1 and AI-2 from V. harveyi mutant strains MM30 (AI-1+, AI-2-) and BB152 (AI-1-, AI-2+) respectively, presented as area under the curve. Presented data are means and SD of three independent experiments, each performed in triplicate. *P < 0.05. Figure S2. Biofilm biomass quantification of V. harveyi wild type BB120, mutant strain MM30 and BB152 biofilms using CV staining. The staining strength is an indication of the amount of biofilm mass formed in AB –media with/without different concentrations of HU-210. Graph represents calculation of biofilm biomass relatively with control. Presented data are means and SD of three independent experiments, each performed in triplicate. *P < 0.05 (DOC 827 kb) [file 12866_2015_499_MOESM1_ESM.doc]

**Supplementary Material**

**Effect of the synthetic cannabinoid HU-210 on quorum sensing and on the production of quorum sensing-mediated virulence factors by *Vibrio harveyi***

Divya Soni^1^

Email: [divyasony.11@gmail.com](mailto:divyasony.11@gmail.com)

Reem Smoum^2^

Email: [reems@ekmd.huji.ac.il](mailto:reems@ekmd.huji.ac.il)

Aviva Breuer^2^

Email: [aviva.breuer@mail.huji.ac.il](mailto:aviva.breuer@mail.huji.ac.il)

Raphael Mechoulam^2^

Email: [raphaelm@ekmd.huji.ac.il](mailto:raphaelm@ekmd.huji.ac.il)

Doron Steinberg^1*^

^*^ Corresponding author

Email: [dorons@ekmd.huji.ac.il](mailto:dorons@ekmd.huji.ac.il)

# ^1^Biofilm Research Laboratory, Institute of Dental Sciences, Faculty of Dental Medicine, Hebrew University-Hadassah Medical Center, Jerusalem, Israel.

# ^2^Institute for Drug Research, Faculty of Medicine, Hebrew University, Jerusalem, Israel.

**Content**

Supplemental Figures:

Fig. S1, Fig. S2


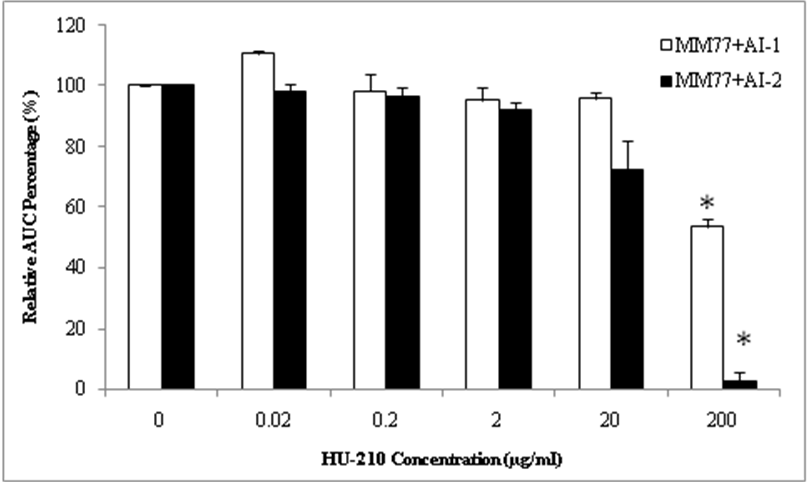


**Suppl. Fig. S1.**

Comparison of relative bioluminescence production by *V. harveyi* MM77 (AI-1^-^, AI-2^-^, sensor-1^+^, sensor-2^+^) with different HU-210 concentrations when simultaneously supplemented with exogenous AI-1 and AI-2 from *V. harveyi* mutant strains MM30 (AI-1^+^, AI-2^-^) and BB152 (AI-1^-^, AI-2^+^) respectively, presented as area under the curve. Presented data are means and SD of three independent experiments, each performed in triplicate. *P < 0.05

**
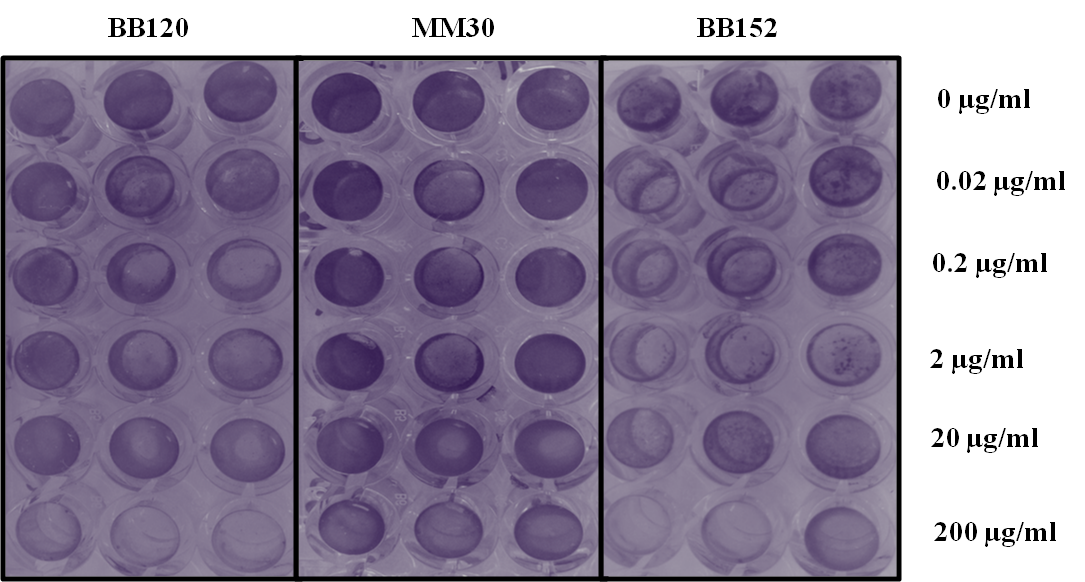
**

**
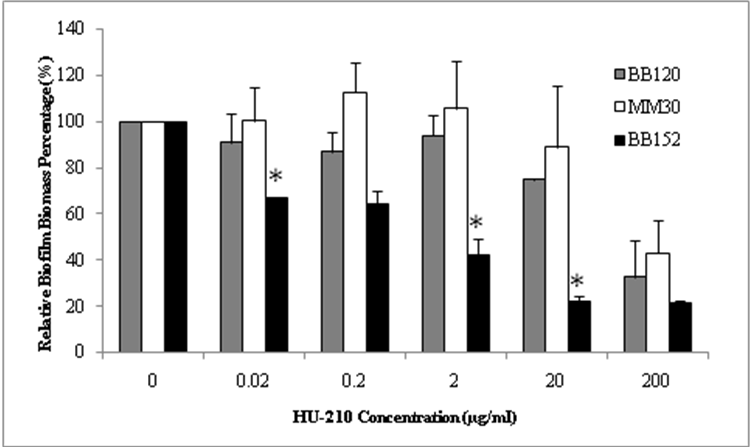
**

**Suppl. Fig. S2.**

Biofilm biomass quantification of *V. harveyi* wild type BB120, mutant strain MM30 and BB152 biofilms using CV staining. The staining strength is an indication of the amount of biofilm mass formed in AB –media with/without different concentrations of HU-210. Graph represents calculation of biofilm biomass relatively with control. Presented data are means and SD of three independent experiments, each performed in triplicate. *P < 0.05
